# Cerebral inflammation is an underlying mechanism of early death in Alzheimer’s disease: a 13-year cause-specific multivariate mortality study

**DOI:** 10.1186/alzrt271

**Published:** 2014-07-07

**Authors:** Katarina Nägga, Carina Wattmo, Yi Zhang, Lars-Olof Wahlund, Sebastian Palmqvist

**Affiliations:** 1 Clinical Memory Research Unit, Department of Clinical Sciences Malmö, Lund University, Malmö, Sweden; 2 Department of Neuroradiology, Karolinska University Hospital, Stockholm, Sweden; 3 Department of Neurobiology, Care Sciences and Society, Section of Clinical Geriatrics, Karolinska Institutet, Karolinska University Hospital, Stockholm, Sweden; 4 Department of Neurology, Skåne University Hospital, Lund SE-221 85, Sweden

## Abstract

**Introduction:**

Although Alzheimer’s disease (AD) is associated with early death, its life expectancy differs greatly between patients. A better understanding of this heterogeneity may reveal important disease mechanisms underlying the malignancy of AD. The aim of this study was to examine the relation between AD pathologies and early death in AD caused by dementia.

**Methods:**

At a memory clinic, 247 referred consecutive patients with AD were monitored during 12.6 ± 1.6 years. Multivariate Cox regression analyses were performed with baseline measures of amyloid beta (Aβ) pathology (*APOE* genotype, cerebrospinal fluid (CSF) Aβ42) tau pathology (CSF phosphorylated tau and total tau), cerebrovascular pathology (white-matter lesions and CSF/serum albumin ratio), neuroinflammatory pathology (CSF soluble vascular cell adhesion molecule-1, sVCAM-1), frontal, temporal, and central brain atrophies, global cognition, sex, and age. Comorbidities and medications also were analyzed. All continuous variables were transformed to *z* scores to compare hazard ratios (HRs) and 95% confidence intervals (CIs).

**Results:**

At follow-up, 89% of the patients had died. The mean survival time was 6.4 ± 3.0 years. The AD pathology that independently predicted an early death caused by dementia was cerebral inflammation (sVCAM-1; HR, 1.32; 95% CI, 1.07–1.64). Other independent predictors were lower global cognition (HR, 0.51; 95% CI, 0.43–0.61), frontal atrophy (HR, 1.38; 95% CI, 1.12–1.70), and medial temporal atrophy (HR, 1.23; 95% CI, 1.02–1.49). When examining death caused by dementia and related causes (vascular diseases and infections), age (HR, 1.23; 95% CI, 1.04–1.46) and cerebrovascular pathology (white-matter lesions: HR, 1.17; 95% CI, 1.01–1.36; and CSF/serum albumin ratio: HR, 1.16; 95% CI, 1.001–1.34) were also significant risk factors in addition to the previous variables. No comorbidity or medication was significant in the specific-cause models.

**Conclusions:**

This is the first study to link neuroinflammation independently to early death in AD and, hence, a rapidly progressing disease. Frontal and medial temporal atrophies and low cognition were also significant predictors. These are probably downstream biomarkers that reflect neuronal degeneration and late-stage disease. Our results suggest that inflammation, and not amyloid or tau pathology, is an independent underlying mechanism in the malignancy of AD.

## Introduction

Alzheimer’s disease (AD) is strongly associated with an early death, compared with the nondemented elderly population [1-5]. Recent data from the United States showed that AD accounts for a population-attributable risk between 5% and 15% on 5-year mortality for ages 65 and older, and represents the fifth leading cause of death in the same age range [6]. Even when adjusting for other diseases that are commonly regarded as causes of death, such as cancer or cardiac diseases, AD remains an independent risk factor for early death [7,8]. The survival from the time of AD diagnosis varies between 3 and 8 years [9,10]. Pneumonia is the most common cause of death in AD in studies using death certificates [11], medical records from the institution where the patient died [12], and studies based on autopsy for the determination of cause of death [13]. However, pneumonia can be considered merely a “symptom” caused by deteriorated health status, dysphagia, malnutrition, or problems with clearing secretions, which is seen in end-stage dementia [11,13-15]. The question, which has been stated before [7], is what mechanisms or pathologies drive the disease rapidly to this end-stage to a greater extent in some patients than in others. A better understanding of the heterogeneity of the life expectancy in AD may highlight important pathologic mechanisms of the malignancy of AD and provide a more accurate prognosis for patients.

AD is associated with different brain pathologies, such as amyloid beta deposition, tau pathology, vascular lesions, and cerebral inflammation [16-18]. The combination of these pathologies differs among patients and can probably explain some of the heterogeneity observed within the spectrum of AD symptoms [19]. Our aim in the present study was to examine whether a certain combination of these pathologies, or the prominence of a specific pathology, can explain the huge difference in life expectancy observed in AD. Therefore, we investigated the predictive ability of AD pathologies and related factors regarding mortality.

## Methods

### Subjects

The patients originated from the Malmö Alzheimer Study [20]. This study consisted of consecutive patients who were referred to the Memory Clinic of the Skåne University Hospital in Malmö, Sweden, because of cognitive complaints. Patients who underwent a lumbar puncture (which was part of the clinical routine) and were diagnosed with probable or possible AD [21] between 1999 and 2003 were included. Physicians with experience in dementia disorders assessed the patients, who underwent computed tomography (CT) or magnetic resonance imaging (MRI) of the brain, lumbar puncture, cognitive tests, and blood analysis.

The Malmö Alzheimer Study consisted of 259 patients. Of these, 12 were excluded from the present study because of missing data; the current study population included 247 patients with a complete data set.

The patients gave their consent for research, and the study was approved by the Regional Ethics Committee in Lund (2012/461 and 2010/401) and the Swedish National Board of Health and Welfare (58744/2012).

### Mortality data

The follow-up consisted of a control whether the patient was alive or not in April 2013, and the follow-up time was defined as the duration from the CT/MRI scan to this control. If the patient was dead, the specific cause of death was obtained from the Swedish Causes of Death Register. The cause of death was determined mostly clinically by an attending responsible physician with knowledge of the patient’s medical history, and, in only a few cases, by autopsy. The causes were specified according to the 10th revision of the International Classification of Disease (ICD-10) [22]. To examine better the pathologies of AD that increase mortality specifically related to AD, the causes of death were divided into three groups based on the diagnoses in the death register.

Dementia

These causes were not restricted to AD (although this was the initial diagnosis), but also included vascular dementia and unspecified dementia (ICD F01, F03, and G30), as some patients acquired other brain pathologies during the time of the study. In these cases, the patient had no other apparent diseases that could have caused death.

Dementia and related causes

In addition to the causes described, this group consisted of cerebrovascular diseases, heart disease, and other vascular diseases; ICD I2X, I4X–I7X), which are risk factors for AD and share the feature of vascular pathology. Infections (most commonly pneumonia, but also infections related to decubitus and unknown infections) were also included in this group (ICD A41, J18, N39), because aspiration, self-neglect, and sedentary lifestyle are very common consequences of late-AD symptoms.

All-cause mortality

This group consisted of all available causes of death including those not related to dementia, such as cancer, gastrointestinal diseases, and chronic obstructive pulmonary disease (COPD).

### Risk factors for mortality: main analysis

The potential risk factors for mortality were chosen based on the known pathologies and biomarkers of AD, and constituted the main analysis. Only baseline variables were used in the study. They were as follows:

#### Amyloid pathology

The 42-amino-acid isoform of amyloid-β_1–42_ (Aβ42) in cerebrospinal fluid (CSF) was analyzed with Luminex xMAP technology to assess amyloid burden [23]. The procedures and analysis of the lumbar puncture and CSF analysis followed the Alzheimer’s Association Flow Chart [24]. Another variable that is associated with amyloid deposition is the apolipoprotein E (*APOE*) ϵ4 allele, especially the presence of two alleles. APOE was therefore entered into the model as a dichotomous variable (that is, presence or absence of ϵ4/ϵ4).

#### Tau pathology

The levels of total tau and tau phosphorylated at Thr181 (P-tau) in the CSF were determined to examine tau pathology. They were analyzed by using the same method as that used to assess Aβ42.

#### Cerebrovascular pathology

White-matter lesions in the left and right frontal and parietal-occipital regions, as well as lesions in the left and right basal ganglia, were assessed by using the Age-Related White Matter Changes (ARWMC) scale [25]. The assessment was performed by S.P. by using CT or MRI scans. The specific procedures, as well as intra- and interrater reliability of this method were published previously [26]. The scores from the different regions were added to obtain a combined score ranging from 0 to 18 points. In addition to the ARWMC scale, the CSF/serum albumin ratio (albumin ratio), which is a marker for the integrity of the blood–brain barrier (BBB), also was calculated.

#### Neuroinflammatory pathology

The CSF levels of the soluble vascular cell-adhesion molecule 1 (sVCAM-1) were analyzed as a measure of cerebral inflammation by using a commercially available quantitative enzyme-linked immunosorbent assay (ELISA) kit (R&D Systems, Minneapolis, MN, USA). This procedure was described previously [20].

#### Regional atrophy

Atrophy is, *per se*, not a pathologic process, but a biomarker of neuronal degeneration, which is a hallmark of AD. Different cerebral areas are affected in AD, depending on disease type and duration [27]. Atrophy was assessed by an experienced radiologist (Y.Z.) by using linear measurements on CT or MRI scans. To include the essential brain regions, the following measures were entered into a Cox regression model: (a) the temporal horn ratio, which measures medial temporal atrophy, (b) Evans ratio, which measures frontal atrophy, and (3) the cella media index, which measures central atrophy. The details of this procedure were published previously [28].

#### Cognition, sex, and age

The Mini-Mental State Examination (MMSE) was used to assess global cognitive function [29]. In addition, sex and age were entered in the models, as both of these characteristics might affect the other independent variables and mortality.

### Concomitant diseases and medications: subanalyses

Concomitant diseases and medications are potentially influential risk factors for the prediction of mortality caused by AD pathology. These variables were not included in the main Cox regression analysis because of their high number. Instead, they were analyzed in separate Cox regression models. Data on current medications and comorbidities were collected from information provided by patients and their relatives and from documentation on diseases and drugs in the patients’ medical records. Several diseases were grouped in larger categories; ischemic heart disease, congestive heart failure, and arrhythmias were named “heart diseases”; all gastrointestinal diseases were grouped together, as well as all pulmonary diseases. Finally, liver and kidney diagnoses were grouped together in one category. Diagnoses with a frequency smaller than 5% were called “all other diseases.”

Current medications were grouped together based on mechanism or treatment indication, except for the category cardiovascular drugs, which included all heart-disease indications and antihypertensive treatment, as these often co-occur, and could not be distinguished from each other.

### Statistics

Cox proportional hazards models were used to estimate separately the effects of the previously mentioned risk factors on the relative risk of time to death (caused by dementia, dementia and related causes, and all-cause mortality). All continuous variables followed a normal distribution and were screened for collinearity by using Pearson correlation; the highest correlation coefficient was seen between tau and P-tau (*r* = 0.77). To facilitate the comparison of hazard ratios (HRs) in the Cox regression analysis, all continuous variables were converted to *z* scores in the main analyses. Thus, the HR corresponded to an increase of 1 standard deviation (SD). This conversion does not affect the model or the significance values of the covariates.

The dichotomous variables were entered into the model if their prevalence was at least 10%, to avoid unstable estimates and a diminished statistical power. This approach did not affect the main analysis, but excluded the following potential predictors from the subanalyses: diabetes, major depression, liver/kidney disease, and medication with antacids, NSAIDs, or hormone therapy. The duration from the date of CT/MRI scan to either death or follow-up (that is, controlling whether the patient was deceased) was used as the time variable. The independent variables were entered by using the stepwise backward likelihood ratio (LR) method with an entry limit of *P* = 0.05 and a removal limit of *P* = 0.051, to avoid including nonsignificant variables in the final model. All analyses were performed with SPSS (IBM Corp. Released 2011. IBM SPSS Statistics for Mac, Version 20.0. Armonk, NY, USA).

## Results

### Demographics

**Figure 1:**
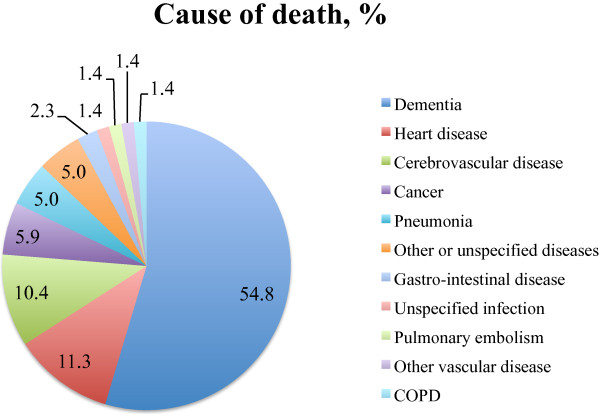
**Cause of death.** Sixty-five different ICD-10 codes were found on the death certificates. These have been grouped into these 11 categories.

The demographics of, and variables analyzed in all 247 patients are shown in Table 1. The mean follow-up time (that is, from CT/MRI scan until the follow-up) was 12.6 years (SD, 1.6 years). At follow-up, 221 (89%) of the 247 patients had died. The mean time to death was 6.4 years (SD, 3.0 years). The causes of death were specified by 65 different ICD-10 codes. These were grouped into 11 different categories (Figure 1). The most common cause was dementia (55%), which was interpreted as an absence of other apparent diseases that could have caused death. Different cerebrovascular, cardiovascular, and other vascular causes of death were the second largest group (23.1%). Infections and cancer were the causes of death in 6.4% and 5.9% of the cases.

**Table 1:** Characteristics (variables entered into the Cox Regression Analysis)

**Variable**	**Mean ± SD (*n*** **= 247)**
Time to death, *n* = 221 (years)	6.4 ± 3.0
Age (years)	75.2 ± 6.1
Sex (female)	69%
MMSE score (0 to 30 points)	21.3 ± 5.1
Temporal horn ratio (medial temporal atrophy)	0.040 ± 0.017
Evans ratio (frontal atrophy)	0.33 ± 0.038
Cella media index (central atrophy)	0.22 ± 0.040
CSF sVCAM-1 (neuroinflammatory pathology, μg/L)	11.7 ± 4.3
ARWMC (cerebrovascular pathology, 0 to 18 points)	3.2 ± 3.4
Albumin ratio (BBB integrity)	0.0075 ± 0.0032
CSF Tau (pg/ml)	619 ± 322
CSF P-tau (pg/ml)	76 ± 31
CSF Aβ42 (pg/ml)	410 ± 99
APOE ϵ4/ϵ4	22%

Aβ, beta-amyloid; APOE, apolipoprotein E; ARWMC, Age-Related White Matter Changes scale; BBB, blood–brain barrier; CSF, cerebrospinal fluid; MMSE, the Mini-Mental State Examination; SD, standard deviation; sVCAM-1, soluble vascular cell adhesion molecule-1.

The concomitant diseases and medications are shown in Table 2. Folate or vitamin B_12_ deficiency was the most common diagnosis (41%), followed by hypertension (32%), gastrointestinal diseases (30%), and heart disease (26%). The most common drugs were antidepressants (43%), followed by cardiovascular drugs (34%) and antipsychotics/sedatives (31%).

**Table 2:** Prevalence of concomitant diseases and medications

**Disease category**	**Prevalence (%)**
Folate or vitamin B_12_ deficiency	41
All other diseases	39
Hypertension	32
Gastrointestinal diseases	30
Heart disease	26
Hypotension	18
Thyroid diseases	17
Hypercholesterolemia	13
Pulmonary diseases	11
Major depression or anxiety disorders	9
Diabetes	8
Liver or kidney diseases	5
**Medications**	
Antidepressants	43
Cardiovascular drugs	34
Platelet-aggregation inhibitors	32
Antipsychotics/sedatives	31
Analgesics (except aspirin and NSAIDs)	13
Thyroxine	12
Serum lipid-reducing agents	12
Antacids	9
Estrogen therapy	7
NSAIDs	2

NSAIDs, nonsteroidal antiinflammatory drugs.

### Prediction of mortality: main analyses

The significant variables for predicting death caused by dementia were the MMSE score (HR, 0.51; 95% CI, 0.43–0.61), Evans ratio (HR, 1.38; 95% CI, 1.12–1.70), sVCAM-1 (HR, 1.32; 95% CI, 1.07–1.64), and temporal horn ratio (HR, 1.23; 95% CI, 1.02–1.49), which are shown in Table 3 (that is, the independent risk factors for death caused by AD were frontal and medial temporal atrophy, high levels of cerebral inflammation, and, most significantly, low cognitive status at baseline). Note that all HRs are based on *z* scores, which allows a head-to-head comparison of the HRs of the variables.

**Table 3:** Risk factors for AD mortality (multivariate Cox regression analyses)

**Cause of death**	**Significant variables**	***P *** **value**	**HR (95% CI)**
Dementia	Cognition	<0.001	0.51 (0.43–0.61)
	Frontal atrophy	0.003	1.38 (1.12–1.70)
	Inflammation	0.010	1.32 (1.07–1.64)
	Medial temporal atrophy	0.032	1.23 (1.02–1.49)
Dementia and related causes	Cognition	<0.001	0.58 (0.50–0.66)
	Frontal atrophy	<0.001	1.33 (1.13–1.57)
	Age	0.019	1.23 (1.04–1.46)
	Inflammation	0.024	1.22 (1.03–1.45)
	Vascular lesions	0.036	1.17 (1.01–1.36)
	BBB integrity	0.048	1.16 (1.001–1.34)
All-cause mortality	Cognition	<0.001	0.59 (0.52–0.67)
	Frontal atrophy	<0.001	1.31 (1.13–1.52)
	Age	0.001	1.28 (1.10–1.49)
	Inflammation	0.005	1.24 (1.07–1.45)

All variables described in Risk factors of mortality (Methods) were entered into each of the three models. The continuous variables were converted to *z* scores. Only significant variables are shown in the table. The following variables were not significant in any of the models: CSF Aβ42 and APOE ϵ4/ϵ4 (amyloid pathology), CSF tau and P-tau (tau pathology), cella media index (central atrophy), and sex. BBB integrity, CSF/serum albumin ratio; cognition, MMSE score; CI, confidence interval; frontal atrophy, Evans ratio; HR, hazard ratio; inflammation, sVCAM-1; medial temporal atrophy, temporal horn ratio; vascular lesions, ARWMC (Age-related White Matter Changes scale).

When predicting dementia- and AD-pathology-related causes of death, the significant variables were Evans ratio (HR, 1.33; 95% CI, 1.13–1.57), sVCAM-1 (HR, 1.22; 95% CI, 1.03–1.45), albumin ratio (HR, 1.16, 95% CI, 1.001–1.34), ARWMC score (HR, 1.17; 95% CI, 1.01–1.36); MMSE score (HR, 0.58; 95% CI, 0.50–0.66), and age (HR, 1.23; 95% CI 1.04–1.46) (Table 3). In addition to frontal atrophy, cerebral inflammation, and cognitive impairment, this model found that cerebrovascular pathology and older age were significant independent predictors for death caused by dementia and related causes. This was probably caused by the vascular origin of most of the AD-related causes of death.

The model for all-cause mortality revealed the following significant variables: Evans ratio (HR, 1.31; 95% CI, 1.13–1.52), age (HR, 1.28; 95% CI, 1.10–1.49), MMSE score (HR, 0.59; 95% CI, 0.52–0.67), and sVCAM-1 (HR, 1.24; 95% CI, 1.07–1.45) (Table 3).

The variables for amyloid (Aβ42 and APOE ϵ4/ϵ4) and tau (total tau and P-tau) pathology were not significant in any of the models (data not shown).

### The effect of comorbidity and medications: subanalyses

No concomitant disease was a risk factor for death caused by dementia (Table 4). Neither could a concomitant disease predict death caused by dementia and related causes; however, older age was a significant risk factor in this model (HR, 1.04; 95% CI, 1.02–1.07). When predicting all-cause mortality, both older age (HR, 1.04; 95% CI, 1.02–1.07) and hypertension (HR, 1.34; 95% CI, 1.01–1.78) were significant variables (Table 4).

**Table 4:** Comorbidity and medication risk factors for mortality (multivariate Cox regression analyses)

**Risk factors (covariates)**	**Cause of death (dependent variable)**	**Significant variables**	***P *** **value**	**HR (95% CI)**
Comorbidities	Dementia	No significant variables		
	Dementia and related causes	Age	<0.001	1.31 (1.12–1.53)
	All-cause mortality	Age	<0.001	1.30 (1.13–1.50)
		Hypertension	0.041	1.34 (1.01–1.78)
Medications	Dementia	No significant variables		
	Dementia and related causes	Age	<0.001	1.30 (1.11–1.52)
	All-cause mortality	Age	0.001	1.27 (1.10–1.47)
		Cardiovascular drugs	0.024	1.38 (1.04–1.83)
		Antipsychotics/sedatives	0.036	1.36 (1.02–1.81)

Age was converted to a *z*-score for comparison with the continuous variables shown in Table 3. All comorbidities and medications with a prevalence ≥10% (Table 2), as well as age and sex, were entered into the Cox regression analyses. Comorbidities and medications were entered separately. HR, hazard ratio.

Similarly, no medications were risk factors for death caused by dementia (Table 4), and older age was a significant risk factor for death caused by dementia and related causes (HR, 1.04; 95% CI, 1.02–1.07). In the all-cause model, the following variables were significant (Table 4): older age (HR, 1.04; 95% CI, 1.02–1.06), antipsychotics/sedatives (HR, 1.36; 95% CI, 1.02–1.82), and cardiovascular drugs (HR, 1.38; 95% CI, 1.04–1.83).

## Discussion

In this 13-year longitudinal study of 247 patients with mild-to-moderate AD, we found that increased cerebral inflammation was the only AD-related pathology that was an independent risk factor for death caused specifically by dementia (Table 3). Other significant risk factors were low cognitive ability, frontal atrophy, and medial temporal lobe atrophy. Tau, amyloid, or cerebrovascular pathology were not significant variables in the Cox regression model for death caused by dementia.

To the best of our knowledge, the present study is the first to show that cerebral inflammation is independently associated with early death in AD. Cerebral inflammation, as measured by CSF sVCAM-1 level, was a risk factor for early death in all mortality groups (Table 3). Previous studies reported that neuroinflammation is involved in the pathogenesis of AD [18,30-33]. It has been suggested that chronic neuroinflammation represents a risk factor for AD in the elderly through an acceleration of senescence in microglia, with a reduction in their neuroprotective function leading to the formation of senile plaques and neurofibrillary tangles [34]. A recent neuropathologic study found that cerebral inflammation measured as glial response is an essential mechanism that determines which of the patients with a high load of tau and amyloid pathology were demented or nondemented [19]. Thus, inflammation might be a key component in the toxicity of β-amyloid, as many individuals have a high burden of amyloid and tau pathology without any clinical symptoms [19]. Furthermore, Forassi *et al*. [35] showed that systemic inflammation is a strong independent risk factor for death in an elderly population.

The other significant risk factors identified (lower cognitive status, frontal atrophy, and medial temporal lobe atrophy) should probably be interpreted as downstream biomarkers, which reflect the disease duration and disease stage. Other studies have also found that low cognitive function and atrophy at baseline are significant risk factors for mortality [36-39]. Frontal atrophy was a stronger predictor than medial temporal atrophy (Table 3), possibly because medial temporal atrophy is an earlier step in the disease process, whereas frontal atrophy occurs later, as an indicator of end-stage dementia [27]. Another explanation could be that AD patients with frontal lobe degeneration constitute a subset of AD with a more rapid disease progression. This assumption is supported by previous results, that revealed that tests of frontal cognitive function, but not those of parietal and temporal function, independently predicted a more rapid AD progression [40].

The results of the subanalyses showed that AD-related factors, but not concomitant diseases or medications, caused early death in AD (Table 4). This was further supported by the fact that neither older age nor male sex predicted specific-cause death (Table 4), even though they are regarded as traditional predictors of death among elderly individuals.

Neuroleptic/sedative drugs were associated with early death in the all-cause model, which was in line with previous studies that showed that psychotic symptoms and/or neuroleptic drugs increase mortality rates [4,41,42]. The mean survival time of 6.4 years found here was in agreement with a large review of 42 studies on AD mortality [43]. Large differences are found in the prevalence of death causes, but overall, pneumonia and dementia have been reported most commonly [3,11,13,44], which differs somewhat from the results of the present study (Figure 1). We believe this can be explained by the manner in which the cause of death was entered on the death certificate, rather than by an actual difference in death causes. If no other specific diagnosis was apparent at the time of death, the attending physician most likely regarded dementia as the cause of death.

One of the strengths of this study was that it constitutes the world’s longest longitudinal study with CSF biomarkers of AD, which ensures a high diagnostic accuracy and a low number of censored cases (patients still alive at follow-up). We also examined predictors of specific-cause death due to dementia, and not only all-cause death, which is essential when trying to map key mechanisms of mortality in AD. Other advantages of our study included the multivariate design of the analysis, which allowed us to examine independent effects of the most common pathologies and hallmarks of AD, and the inclusion of patients from a routine clinical setting.

We chose to measure cerebral inflammation by using sVCAM-1 levels in the CSF, based on previous validating studies [45-50]. sVCAM-1 is a cell-surface molecule that regulates leukocyte migration and is activated by proinflammatory factors such as cytokines and interleukins [49]. Moreover, it has been associated with glial activation (expressed on astrocytes) [47]. Numerous studies have associated increased levels of sVCAM-1 with several neuroinflammatory conditions, and it has been significantly correlated with inflammatory cerebral lesions assessed by using contrast MRI [45-50]. sVCAM-1 is also a well-proven marker of endothelial dysfunction and has been associated with atherosclerosis [51] and small-vessel disease [52]. However, based on our results, we believe that sVCAM-1 is related more directly to neuroinflammation, rather than to cerebrovascular pathology, as sVCAM-1, not vascular lesions and BBB integrity, was an independent significant variable in the main analysis performed to predict death caused by dementia (Table 3). Vascular lesions and BBB integrity were, however, separately significant when examining death also caused by cerebrovascular disorders, which validates them as adequate markers of vascular pathology in this data set.

To warrant our conclusions, the results of this exploratory study should be tested in larger longitudinal cohorts including additional factors that are associated with shorter survival (for example, diabetes) and using other markers of inflammation such as IL-1 on either the protein or mRNA level.

## Conclusions

We found that cerebral inflammation measured by using sVCAM-1 levels in the CSF was an independent risk factor for early death in AD. A more-advanced disease stage was also a risk factor, as assessed by using the significantly independent variables of global cognition and medial temporal and frontal atrophy. The biomarkers of tau or amyloid were not significant risk factors, and cerebrovascular pathology was only a significant risk factor when including death caused by vascular disorders and infections. This is the first study of its kind, and further research is needed to confirm these findings. As early death is linked to a rapid disease progression [53], our results could have implications for estimating the malignancy or toxicity of the AD process, and for selecting correct targets in future clinical trials of disease-modifying therapies.

## Abbreviations

APOE: Apolipoprotein E; ARWMC: age-related white-matter changes; Aβ: amyloid beta; Aβ42: the 42-amino-acid isoform of amyloid-β_1–42_; BBB: blood–brain barrier; COPD: chronic obstructive pulmonary disease; CSF: cerebrospinal fluid; CT: computed tomography; ELISA: enzyme-linked immunosorbent assay; HR: hazard ratio; ICD-10: the tenth revision of the International Classification of Disease; LR: likelihood ratio; MMSE: Mini-Mental State Examination; MRI: magnetic resonance imaging; P-tau: Tau phosphorylated at Thr181; SD: standard deviation; sVCAM-1: soluble vascular cell-adhesion molecule 1.

## Competing interests

The authors declare that they have no competing interests.

## Authors’ contributions

KN, data collection, writing of manuscript draft, critical manuscript revision, and final approval of the manuscript. CW, review of statistical analysis, critical manuscript revision, and final approval of the manuscript. YZ, assessment of atrophy measures, critical manuscript revision, and final approval of the manuscript. LOW, supervision, critical manuscript revision, and final approval of the manuscript. SP, conception and design, supervision, data collection and analysis, assessment of cerebrovascular lesions, writing of manuscript draft, critical manuscript revision, and final approval of the manuscript. All authors read and approved the final manuscript.

## References

[B1] Jagger C Andersen K Breteler MM Copeland JR Helmer C Baldereschi M Fratiglioni L Lobo A Soininen H Hofman A Launer LJ Prognosis with dementia in Europe: a collaborative study of population-based cohorts: Neurologic Diseases in the Elderly Research Group Neurology 2000 54 S16 S20 10854356

[B2] Lonnroos E Kyyronen P Bell JS van der Cammen TJ Hartikainen S Risk of death among persons with Alzheimer’s disease: a national register-based nested case–control study J Alzheimers Dis 2013 33 157 164 22914589 10.3233/JAD-2012-120808

[B3] Ganguli M Dodge HH Shen C Pandav RS DeKosky ST Alzheimer disease and mortality: a 15-year epidemiological study Arch Neurol 2005 62 779 784 15883266 10.1001/archneur.62.5.779

[B4] Tsai PH Chen SP Lin KN Wang PN Wang HC Liu CY Hong CJ Liu HC Survival of ethnic Chinese with Alzheimer’s disease: a 5-year longitudinal study in Taiwan J Geriatr Psychiatry Neurol 2007 20 172 177 17712101 10.1177/0891988707301864

[B5] Arrighi HM Neumann PJ Lieberburg IM Townsend RJ Lethality of Alzheimer disease and its impact on nursing home placement Alzheimer Dis Assoc Disord 2010 24 90 95 19568155 10.1097/WAD.0b013e31819fe7d1

[B6] Association As Alzheimer’s disease facts and figures Alzheimers Dement 2012 8 131 168 22404854 10.1016/j.jalz.2012.02.001

[B7] Aguero-Torres H Fratiglioni L Guo Z Viitanen M Winblad B Mortality from dementia in advanced age: a 5-year follow-up study of incident dementia cases J Clin Epidemiol 1999 52 737 743 10465318 10.1016/s0895-4356(99)00067-0

[B8] Eaker ED Vierkant RA Mickel SF Predictors of nursing home admission and/or death in incident Alzheimer’s disease and other dementia cases compared to controls: a population-based study J Clin Epidemiol 2002 55 462 468 12007549 10.1016/s0895-4356(01)00498-x

[B9] Brookmeyer R Corrada MM Curriero FC Kawas C Survival following a diagnosis of Alzheimer disease Arch Neurol 2002 59 1764 1767 12433264 10.1001/archneur.59.11.1764

[B10] Helzner EP Scarmeas N Cosentino S Tang MX Schupf N Stern Y Survival in Alzheimer disease: a multiethnic, population-based study of incident cases Neurology 2008 71 1489 1495 18981370 10.1212/01.wnl.0000334278.11022.42 PMC2843528

[B11] Thomas BM Starr JM Whalley LJ Death certification in treated cases of presenile Alzheimer’s disease and vascular dementia in Scotland Age Ageing 1997 26 401 406 9351485 10.1093/ageing/26.5.401

[B12] Ueki A Shinjo H Shimode H Nakajima T Morita Y Factors associated with mortality in patients with early-onset Alzheimer’s disease: a five-year longitudinal study Int J Geriatr Psychiatry 2001 16 810 815 11536348 10.1002/gps.419

[B13] Brunnstrom HR Englund EM Cause of death in patients with dementia disorders Eur J Neurol 2009 16 488 492 19170740 10.1111/j.1468-1331.2008.02503.x

[B14] Mölsä PK Marttila RJ Rinne UK Survival and cause of death in Alzheimer’s disease and multi-infarct dementia Acta Neurol Scand 1986 74 103 107 3776457 10.1111/j.1600-0404.1986.tb04634.x

[B15] Ryan D Death in dementia: a study of causes of death in dementia patients and their spouses Int J Geriatr Psychiatry 1992 7 465 472

[B16] Iadecola C The overlap between neurodegenerative and vascular factors in the pathogenesis of dementia Acta Neuropathol 2010 120 287 296 20623294 10.1007/s00401-010-0718-6 PMC3001188

[B17] Jack CR Jr Knopman DS Jagust WJ Petersen RC Weiner MW Aisen PS Shaw LM Vemuri P Wiste HJ Weigand SD Lesnick TG Pankratz VS Donohue MC Trojanowski JQ Tracking pathophysiological processes in Alzheimer’s disease: an updated hypothetical model of dynamic biomarkers Lancet Neurol 2013 12 207 216 23332364 10.1016/S1474-4422(12)70291-0 PMC3622225

[B18] Zotova E Nicoll JA Kalaria R Holmes C Boche D Inflammation in Alzheimer’s disease: relevance to pathogenesis and therapy Alzheimers Res Ther 2010 2 1 20122289 10.1186/alzrt24 PMC2874260

[B19] Perez-Nievas BG Stein TD Tai HC Dols-Icardo O Scotton TC Barroeta-Espar I Fernandez-Carballo L de Munain EL Perez J Marquie M Serrano-Pozo A Frosch MP Lowe V Parisi JE Petersen RC Ikonomovic MD Lopez OL Klunk W Hyman BT Gomez-Isla T Dissecting phenotypic traits linked to human resilience to Alzheimer’s pathology Brain 2013 136 2510 2526 23824488 10.1093/brain/awt171 PMC3722351

[B20] Nielsen HM Londos E Minthon L Janciauskiene SM Soluble adhesion molecules and angiotensin-converting enzyme in dementia Neurobiol Dis 2007 26 27 35 17270454 10.1016/j.nbd.2006.11.011

[B21] McKhann G Drachman D Folstein M Katzman R Price D Stadlan EM Clinical diagnosis of Alzheimer’s disease: report of the NINCDS-ADRDA Work Group under the auspices of Department of Health and Human Services Task Force on Alzheimer’s Disease Neurology 1984 34 939 944 6610841 10.1212/wnl.34.7.939

[B22] World Health Organization Manual of the International Statistical Classification of Diseases and Health Related Problems. 10th revision 1992 Geneva, Switzerland: World Health Organization

[B23] Olsson A Vanderstichele H Andreasen N De Meyer G Wallin A Holmberg B Rosengren L Vanmechelen E Blennow K Simultaneous measurement of beta-amyloid (1–42), total tau, and phosphorylated tau (Thr181) in cerebrospinal fluid by the xMAP technology Clin Chem 2005 51 336 345 15563479 10.1373/clinchem.2004.039347

[B24] Blennow K Hampel H Weiner M Zetterberg H Cerebrospinal fluid and plasma biomarkers in Alzheimer disease Nat Rev Neurol 2010 6 131 144 20157306 10.1038/nrneurol.2010.4

[B25] Wahlund L Barkhof F Fazekas F Bronge L Augustin M Sjögren M Wallin A Ader H Leys D Pantoni L Pasquier F Erkinjuntti T Scheltens P A new rating scale for age-related white matter changes applicable to MRI and CT Stroke 2001 32 1318 1322 11387493 10.1161/01.str.32.6.1318

[B26] Palmqvist S Sarwari A Wattmo C Bronge L Zhang Y Wahlund LO Nagga K Association between subcortical lesions and behavioral and psychological symptoms in patients with Alzheimer’s disease Dement Geriatr Cogn Disord 2011 32 417 423 22343788 10.1159/000335778

[B27] Whitwell JL Progression of atrophy in Alzheimer’s disease and related disorders Neurotox Res 2010 18 339 346 20352396 10.1007/s12640-010-9175-1

[B28] Zhang Y Londos E Minthon L Wattmo C Liu H Aspelin P Wahlund LO Usefulness of computed tomography linear measurements in diagnosing Alzheimer’s disease Acta Radiol 2008 49 91 97 18210318 10.1080/02841850701753706

[B29] Folstein MF Folstein SE McHugh PR “Mini-mental state:” a practical method for grading the cognitive state of patients for the clinician J Psychiatr Res 1975 12 189 198 1202204 10.1016/0022-3956(75)90026-6

[B30] Eikelenboom P Hoozemans JJ Veerhuis R van Exel E Rozemuller AJ van Gool WA Whether, when and how chronic inflammation increases the risk of developing late-onset Alzheimer’s disease Alzheimers Res Ther 2012 4 15 22647384 10.1186/alzrt118 PMC3506930

[B31] Krstic D Knuesel I Deciphering the mechanism underlying late-onset Alzheimer disease Nat Rev Neurol 2013 9 25 34 23183882 10.1038/nrneurol.2012.236

[B32] McGeer PL McGeer EG Local neuroinflammation and the progression of Alzheimer’s disease J Neurovirol 2002 8 529 538 12476347 10.1080/13550280290100969

[B33] Tan ZS Seshadri S Inflammation in the Alzheimer’s disease cascade: culprit or innocent bystander? Alzheimers Res Ther 2010 2 6 20388190 10.1186/alzrt29 PMC2876784

[B34] Krstic D Madhusudan A Doehner J Vogel P Notter T Imhof C Manalastas A Hilfiker M Pfister S Schwerdel C Riether C Meyer U Knuesel I Systemic immune challenges trigger and drive Alzheimer-like neuropathology in mice J Neuroinflamm 2012 9 151 10.1186/1742-2094-9-151 PMC3483167 22747753

[B35] Forasassi C Golmard JL Pautas E Piette F Myara I Raynaud-Simon A Inflammation and disability as risk factors for mortality in elderly acute care patients Arch Gerontol Geriatr 2009 48 406 410 18456352 10.1016/j.archger.2008.03.011

[B36] Henneman WJ Sluimer JD Cordonnier C Baak MM Scheltens P Barkhof F van der Flier WM MRI biomarkers of vascular damage and atrophy predicting mortality in a memory clinic population Stroke 2009 40 492 498 19109551 10.1161/STROKEAHA.108.516286

[B37] Hotte SD Lankers D Kissler S Freyberger HJ Schroder SG MMSE for survival prognostics in dementia Psychiatr Prax 2010 37 78 83 20183772 10.1055/s-0029-1223473

[B38] Larson EB Shadlen MF Wang L McCormick WC Bowen JD Teri L Kukull WA Survival after initial diagnosis of Alzheimer disease Ann Intern Med 2004 140 501 509 15068977 10.7326/0003-4819-140-7-200404060-00008

[B39] Walsh JS Welch HG Larson EB Survival of outpatients with Alzheimer-type dementia Ann Intern Med 1990 113 429 434 2386336 10.7326/0003-4819-113-6-429

[B40] Musicco M Salamone G Caltagirone C Cravello L Fadda L Lupo F Mosti S Perri R Palmer K Neuropsychological predictors of rapidly progressing patients with Alzheimer’s disease Dement Geriatr Cogn Disord 2010 30 219 228 20838048 10.1159/000319533

[B41] Scarmeas N Brandt J Albert M Hadjigeorgiou G Papadimitriou A Dubois B Sarazin M Devanand D Honig L Marder K Bell K Wegesin D Blacker D Stern Y Delusions and hallucinations are associated with worse outcome in Alzheimer disease Arch Neurol 2005 62 1601 1608 16216946 10.1001/archneur.62.10.1601 PMC3028538

[B42] Schneider LS Dagerman KS Insel P Risk of death with atypical antipsychotic drug treatment for dementia: meta-analysis of randomized placebo-controlled trials JAMA 2005 294 1934 1943 16234500 10.1001/jama.294.15.1934

[B43] Brodaty H Seeher K Gibson L Dementia time to death: a systematic literature review on survival time and years of life lost in people with dementia Int Psychogeriatr 2012 24 1034 1045 22325331 10.1017/S1041610211002924

[B44] Wachterman M Kiely DK Mitchell SL Reporting dementia on the death certificates of nursing home residents dying with end-stage dementia JAMA 2008 300 2608 2610 19066379 10.1001/jama.2008.768 PMC2670182

[B45] Jaber SM Hamed EA Hamed SA Adhesion molecule levels in serum and cerebrospinal fluid in children with bacterial meningitis and sepsis J Pediatr Neurosci 2009 4 76 85 21887188 10.4103/1817-1745.57326 PMC3162794

[B46] James WG Bullard DC Hickey MJ Critical role of the alpha 4 integrin/VCAM-1 pathway in cerebral leukocyte trafficking in lupus-prone MRL/fas(lpr) mice J Immunol 2003 170 520 527 12496439 10.4049/jimmunol.170.1.520

[B47] Oh JW Van Wagoner NJ Rose-John S Benveniste EN Role of IL-6 and the soluble IL-6 receptor in inhibition of VCAM-1 gene expression J Immunol 1998 161 4992 4999 9794436

[B48] Rieckmann P Altenhofen B Riegel A Kallmann B Felgenhauer K Correlation of soluble adhesion molecules in blood and cerebrospinal fluid with magnetic resonance imaging activity in patients with multiple sclerosis Mult Scler 1998 4 178 182 9762670 10.1177/135245859800400317

[B49] Singh RJ Mason JC Lidington EA Edwards DR Nuttall RK Khokha R Knauper V Murphy G Gavrilovic J Cytokine stimulated vascular cell adhesion molecule-1 (VCAM-1) ectodomain release is regulated by TIMP-3 Cardiovasc Res 2005 67 39 49 15949468 10.1016/j.cardiores.2005.02.020

[B50] Uzawa A Mori M Masuda S Kuwabara S Markedly elevated soluble intercellular adhesion molecule 1, soluble vascular cell adhesion molecule 1 levels, and blood–brain barrier breakdown in neuromyelitis optica Arch Neurol 2011 68 913 917 21747031 10.1001/archneurol.2011.148

[B51] Cybulsky MI Iiyama K Li H Zhu S Chen M Iiyama M Davis V Gutierrez-Ramos JC Connelly PW Milstone DS A major role for VCAM-1, but not ICAM-1, in early atherosclerosis J Clin Invest 2001 107 1255 1262 11375415 10.1172/JCI11871 PMC209298

[B52] Rouhl RP Damoiseaux JG Lodder J Theunissen RO Knottnerus IL Staals J Henskens LH Kroon AA de Leeuw PW Tervaert JW van Oostenbrugge RJ Vascular inflammation in cerebral small vessel disease Neurobiol Aging 2012 33 1800 1806 21601314 10.1016/j.neurobiolaging.2011.04.008

[B53] Hui JS Wilson RS Bennett DA Bienias JL Gilley DW Evans DA Rate of cognitive decline and mortality in Alzheimer’s disease Neurology 2003 61 1356 1361 14638955 10.1212/01.wnl.0000094327.68399.59

